# MiRNAs Correlate with HLA Expression in Uveal Melanoma: Both Up- and Downregulation Are Related to Monosomy 3

**DOI:** 10.3390/cancers13164020

**Published:** 2021-08-10

**Authors:** Zahra Souri, Annemijn P. A. Wierenga, Emine Kiliç, Erwin Brosens, Stefan Böhringer, Wilma G. M. Kroes, Robert M. Verdijk, Pieter A. van der Velden, Gregorius P. M. Luyten, Martine J. Jager

**Affiliations:** 1 Department of Ophthalmology, LUMC, 2333ZA Leiden, The Netherlands; z.souri@lumc.nl (Z.S.); a.p.a.wierenga@lumc.nl (A.P.A.W.); p.a.van_der_velden@lumc.nl (P.A.v.d.V.); g.p.m.luyten@lumc.nl (G.P.M.L.); 2 Department of Ophthalmology, Erasmus MC, 3015 GD Rotterdam, The Netherlands; e.kilic@erasmusmc.nl; 3 Department of Clinical Genetics, Erasmus MC, 3000 CA Rotterdam, The Netherlands; e.brosens@erasmusmc.nl; 4 Department of Medical Statistics, LUMC, 2300 RC Leiden, The Netherlands; s.boehringer@lumc.nl; 5 Department of Clinical Genetics, LUMC, 2300 RC Leiden, The Netherlands; w.g.m.kroes@lumc.nl; 6 Department of Pathology, LUMC, 2333ZA Leiden, The Netherlands; r.m.verdijk@lumc.nl; 7 Department of Pathology, Erasmus MC, 3015 GD Rotterdam, The Netherlands

**Keywords:** eye disease, uveal melanoma, oncology, inflammation, miRNA, HLA, TAP1, monosomy 3, BAP1

## Abstract

**Simple Summary:**

Uveal melanoma (UM) is a rare ocular malignancy that often gives rise to metastases. Tumours with an inflammatory phenotype have an especially bad prognosis. As an increased HLA expression and the presence of tumour-infiltrating lymphocytes and macrophages may be regulated by miRNAs, we set out to investigate whether any miRNAs are associated with inflammatory parameters in this malignancy. Some miRNAs were increased in UM with a high HLA expression and high T cell numbers, while others were decreased, showing two opposing patterns; however, both patterns were related to the tumour’s chromosome 3/BAP1 status. We conclude that specific miRNAs are related to the inflammatory phenotype and that these are differentially expressed between disomy 3/BAP1-positive versus monosomy 3/BAP1-negative UM.

**Abstract:**

MicroRNAs are known to play a role in the regulation of inflammation. As a high HLA Class I expression is associated with a bad prognosis in UM, we set out to determine whether any miRNAs were related to a high HLA Class I expression and inflammation. We also determined whether such miRNAs were related to the UM’s genetic status. The expression of 125 miRNAs was determined in 64 primary UM from Leiden. Similarly, the mRNA expression of HLA-A, HLA-B, TAP1, BAP1, and immune cell markers was obtained. Expression levels of 24 of the 125 miRNAs correlated with expression of at least three out of four HLA Class I probes. Four miRNAs showed a positive correlation with HLA expression and infiltration with leukocytes, 20 a negative pattern. In the first group, high miRNA levels correlated with chromosome 3 loss/reduced BAP1 mRNA expression, in the second group low miRNA levels. The positive associations between miRNA-22 and miRNA-155 with HLA Class I were confirmed in the TCGA study and Rotterdam cohort, and with TAP1 in the Rotterdam data set; the negative associations between miRNA-125b2 and miRNA-211 and HLA-A, TAP1, and CD4 were confirmed in the Rotterdam set. We demonstrate two patterns: miRNAs can either be related to a high or a low HLA Class I/TAP1 expression and the presence of infiltrating lymphocytes and macrophages. However, both patterns were associated with chromosome 3/BAP1 status, which suggests a role for BAP1 loss in the regulation of HLA expression and inflammation in UM through miRNAs.

## 1. Introduction

Uveal melanoma (UM) is the most common type of primary intraocular malignancy in adults and carries a high risk of metastases. Tumours with monosomy of chromosome 3 (M3), gain of 8q [1,2,3,4], a mutation in the BAP1 (BRCA1-associated protein-1) gene [5,6], or a class 2 gene expression profile [7,8,9] are more prone to develop metastases. Tumours that have these high-risk characteristics often show an inflammatory phenotype [10,11], characterized by the presence of high numbers of tumour-infiltrating lymphocytes and macrophages [12,13], and a high expression of the HLA Class I and II antigens [14,15,16]. HLA Class I molecules are essential for the presentation of processed tumour antigens to cytotoxic T cells and are therefore important for immunotherapeutic approaches. However, while a high HLA expression might theoretically make the tumour cells more susceptible to killing by cytotoxic T cells, it may decrease their susceptibility to NK (natural killer) cell-mediated lysis, which is probably especially relevant during hematogeneous tumour cell migration from the eye to the liver [17,18].

Although there are effective treatments available for primary UM (radio-active plaque, proton beam therapy, stereotactic irradiation), a treatment to cure metastases is still missing and survival of high-risk cases has not improved during the last 50 years [19,20,21,22]; it is therefore important to identify new potential therapies to treat metastases of this disease. Targeting different types of RNA might be an approach.

MiRNAs are short noncoding RNAs, usually with a length of 17–22 base pairs. By binding to 3ʹUTR regions of complimentary mRNAs, they either stimulate or delay translation of specific genes and thereby play an important role in gene regulation [23]. In recent years, miRNAs have been identified as new players in tumorigenesis and metastases formation, and they have been associated with angiogenesis [24,25], the induction of epithelial-to-mesenchymal transition [26] and increased proliferation [27,28,29]. Several miRNAs have been identified to play a role in tumorigenesis of UM [30], and differences in miRNA expression occur between UM tumours with a low or high metastatic risk [31,32,33,34]. Smit reported that miRNAs were potential actors for the progression of metastasis in UM: 13 out of 423 mature miRNAs, which were expressed in UM samples, were differentially expressed between low, intermediate, and high-risk UM [35]. MiRNAs may act as oncomirs leading to cell growth and invasion [36,37], or as tumour suppressors, having anti-tumour activity [38].

Inflammation is a bad prognostic sign in UM, but it is as yet unclear how this inflammation is regulated. The presence of tumour-associated macrophages (TAMs) has been associated with loss of one chromosome 3 (M3, monosomy 3) and gain of chromosome 8q, while both TAMs as well as tumour-infiltrating lymphocytes (TILs) are increased in UM with loss of chromosome 3/BAP1 [10,13,39].

**Figure 1:**
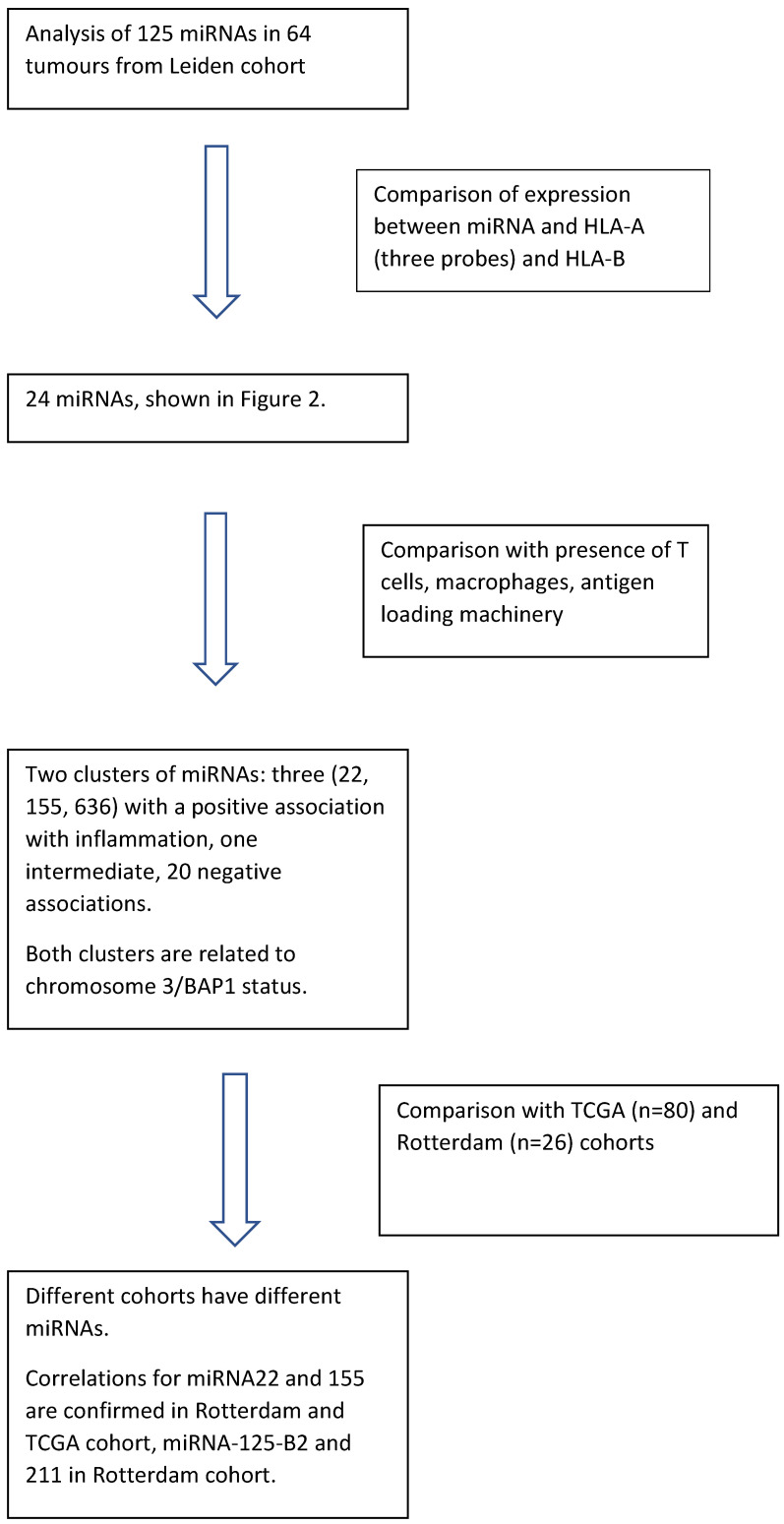
Schedule for analyses.

As miRNAs may influence inflammation, we analysed 125 miRNAs in 64 UM samples and determined any association with HLA Class I expression and the presence of an infiltrate; we furthermore investigated whether the levels of inflammation-associated miRNAs were related to the tumour genetic status. Outcomes were compared with data from the Department of Ophthalmology, ErasmusMC, Rotterdam, The Netherlands [35], and TCGA data [11] (See Schedule in Figure 1).

## 2. Materials and Methods

### 2.1. Study Population Leiden Cohort

Tumour material was obtained from 64 eyes that underwent an enucleation for UM between 1999 and 2008 at the Leiden University Medical Center (LUMC) in Leiden, The Netherlands. Characteristics of the cohort have been added as Table S1. 51% of the patients were male and 49% were female. The mean age at the time of enucleation was 61 years. The mean follow-up time (defined as the time period between enucleation and either date of last follow-up or death) was 83 months (range 2 to 229 months). Follow-up was updated in 2020. At the end of follow up, 17 (27%) patients were alive, 37 (58%) patients had died because of metastasis, four (6%) had died because of other causes and six (9%) had died but the cause of death was unknown.

### 2.2. Chromosome Status

DNA isolation was performed on frozen tumour tissue using the QIAmp DNA Mini kit for single nucleotide polymorphism (SNP) detection (Qiagen, Venlo, The Netherlands). Chromosome 3 abnormality was detected by SNP microarray analysis using an Affymetrix 250K_NSP (Affymetrix, Santa Clara, CA, USA) array as described previously [4,13].

### 2.3. Gene Expression

RNA isolation was performed on frozen tumour tissue using the RNeasy mini kit (Qiagen, Venlo, The Netherlands). Gene expression was determined with the Illumina HT12v4 array (Illumina, Inc., San Diego, CA, USA) for 125 microRNA, HLA Class I (HLA-A, HLA-B), HLA Class I Regulatory Factors and the Antigen-Loading Machinery (CIITA, NLRC5, IRF1, IRF8 and TAP1), Immune markers (CD3, CD4, CD8, CD68, CD163), and BAP1 as described previously [40].

### 2.4. Rotterdam Cohort

The 26 cases analysed from the ErasmusMC were described by Smit [35]. Both miRNA and mRNA libraries were sequenced with the Ion Proton sequencer. Readtrimming using Cutadapt Version 3.4 [41] alignment to the hg19 reference genome using HISAT2 Version 2.1.0 [42]. Aligned reads were counted using htseq-count Version 0.9.1 [43] mirBasev20, Homo_Sapiens.GRCh37.75) and normalized using DESeq2.

### 2.5. TCGA Data

MiRNA expression data of 80 UM samples were retrieved from the repository of the Genomic Data Commons Data Portal (https://portal.gdc.cancer.gov) [11].

### 2.6. Statistical Analysis

Data were analysed with SPSS software version 22.0 (SPSS, nc., Chicago, IL, USA). Graphs were obtained using GraphPad Prism version 5.0 for windows (GraphPad Software, La Jolla, CA, USA). Spearman correlation was performed in order to make correlations between non-parametric data. The Mann-Whitney U test was used to compare non-normal groups. Kaplan-Meier survival curves were made and the log rank test was used to determine significance.

We used R-package heatmaply to produce the heatmaps. We use 1-cor(X, Y) to define the distance between mRNA X and Y to be used for the hiarchical clustering. The linkage function was average linkage. R version 3.6.1 was used.

### 2.7. Institutional Review Board Statement and Informed Consent

This project was approved by the METC of the LUMC (B14.003/SH/sh Approval Biobank OOG-2 “Oogtumoren (of een verdenking hierop)”). The research adhered to Dutch law and the tenets of the Declaration of Helsinki (World Medical Association of Declaration 2013; ethical principles for medical research involving human subjects). Informed consent was obtained from all subjects involved in the study.

## 3. Results

### 3.1. MiRNAs and HLA Class I

As HLA antigens are one of the characteristics of high-risk UM and may play an important role in immunotherapy, it is important to know how their expression is regulated. As a relation between miRNAs and HLA expression has been observed in other malignancies, we determined whether we could identify miRNAs that were related to HLA expression, one of the markers of the inflammatory phenotype of UM.

**Figure 2:**
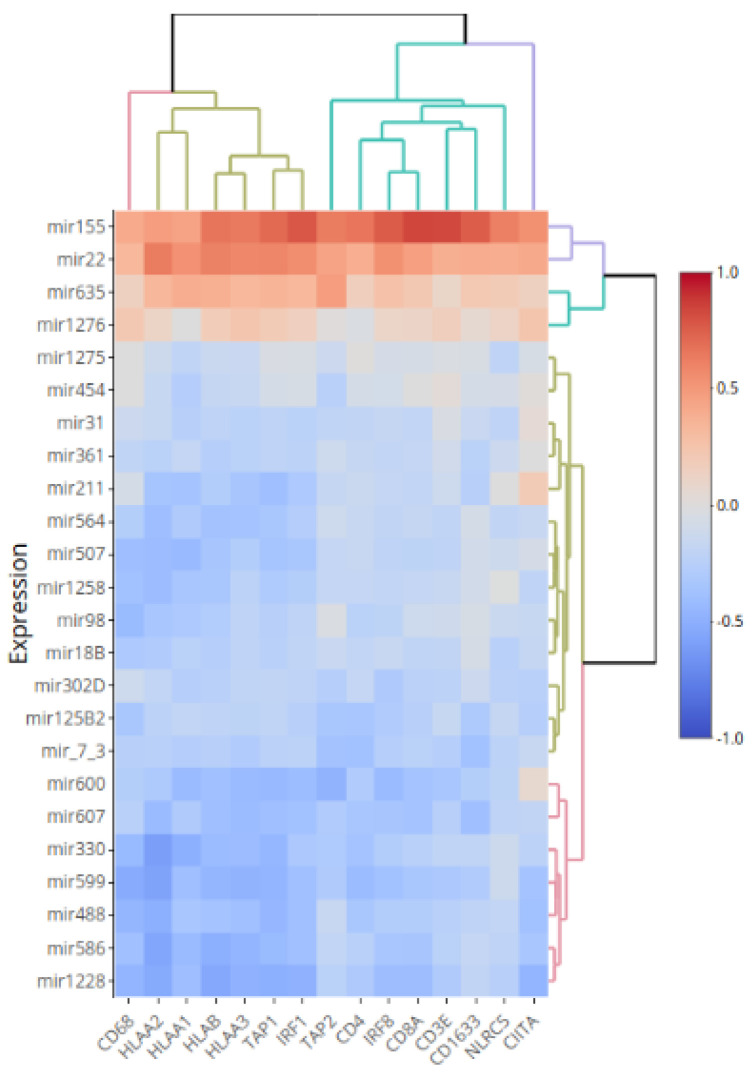
Heat map of the correlations between the expression levels of 24 miRNA expression with the mRNA expression of three HLA-A probes (probes HLA-A pr1, HLA-A pr2 and HLA-A pr3) and HLA-B, several regulators of HLA, members of the Antigen Loading Machinery, and T cell and macrophage markers. An analysis of 64 Uveal Melanoma was used to obtain the correlations. Red indicates a positive correlation and blue a negative correlation.

Using a set of 64 UM from the LUMC, we compared the expression levels of miRNAs with expression of HLA Class I, using four HLA Class I probes (three for HLA-A, one for HLA-B). Of the 125 studied miRNAs, 24 showed a correlation with at least three of the four HLA probes (Table S2). Two different patterns were observed: four miRNAs (miR-155, miR-22, miR-635 and miR-1276) showed positive correlations with at least three of the four HLA Class I probes and 20 miRNAs showed negative correlations with at least three of the four HLA Class I probes. For these 24 miRNAs, we compared the miRNA expression levels not only with HLA-A and -B expression, but also with the expression of probes for HLA regulators (TAP1, TAP2, IRF1, IRF8, CIITA, NLRC5) [40], and markers of infiltrating T cells and macrophages (see Figure 2 and Figure S1 for TAP1). A clear pattern emerged, with the same four miRNAs showing positive correlations with most markers, and the other twenty mainly showing negative correlations.

In order to validate our findings, we investigated the association between miRNAs, HLA Class I and TAP1 expression in a cohort of 26 UM from the Rotterdam ErasmusMC (Table S4). Of the 47 miRs, only eleven showed a high enough expression to be able to use them in a comparison with HLA mRNA expression data from the same cohort. MiR-155 showed a positive correlation with TAP1 (*R* = 0.502, *p* = 0.02). MiR-211 showed a negative correlation with HLA-A, and TAP1, and miR-125 with HLA-A, HLA-B and TAP1. MiR-635 and miR-1276 were not available in this set. Of the other miRs that were in both sets, miR-18B, miR-31, miR-98, miR-361, miR-454, and miR-507 did not show any significant correlations with HLA-A, HLA-B, or TAP1 expression in the Rotterdam data.

We then screened the data of the TCGA, where only three of the 24 miRNA of interest were identified: miR-155 and miR-22 were positively correlated with HLA-A (*R* = 0.630, *p* < 0.001; *R* = 0.328, *p* = 0.003, respectively) and HLA-B (*R* = 0.694, *p* < 0.001; *R* = 0.396, *p* < 0.001), while miR-98 did not show a significant association with HLA-A nor HLA-B (*R* = 0.113, *p* = 0.32; *R* = 0.054, *p* = 0.63).

### 3.2. MiRNA Expression and Correlation with TILs and TAMs in Uveal Melanoma

As not only HLA expression but also the presence of an infiltrate is an important characteristic of an inflammatory phenotype, we analyzed whether the association was not only with HLA expression but also with the presence of TILs and TAMs. Out of the 101 miRNAs that were not associated with HLA expression, only three showed significant correlations with the presence of TILs and TAMs, indicating that HLA expression and a leukocyte infiltrate are strongly related (Table S3). We then focused on the 24 miRNAs which were correlated with HLA Class I expression. 

**Figure 3:**
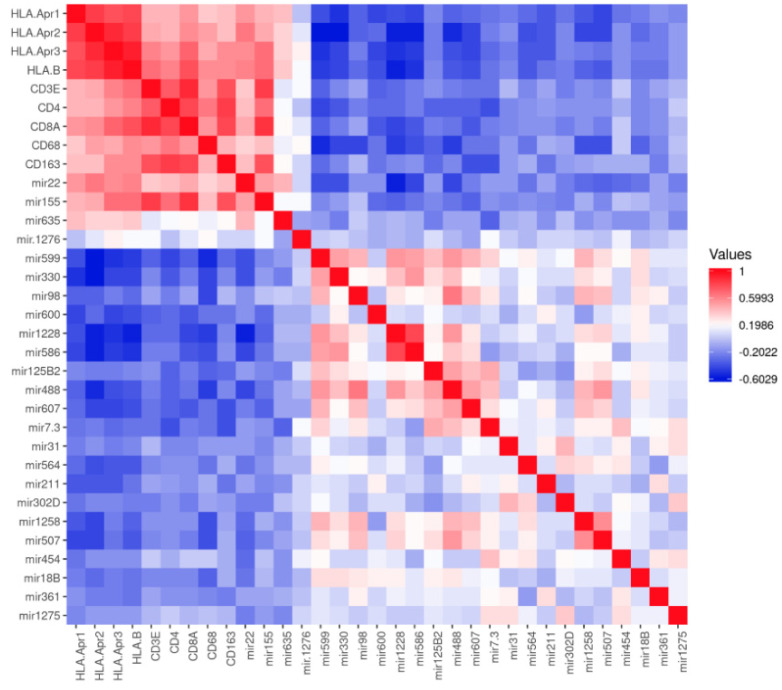
Expression of miRNAs was compared to the mRNA expression of HLA Class I and markers of infiltrate. Relation between miRNAs and mRNA levels from 64 Uveal melanoma are shown in a pairwise correlation matrix. Red indicates a positive correlation, blue a negative correlation.

**Figure 4:**
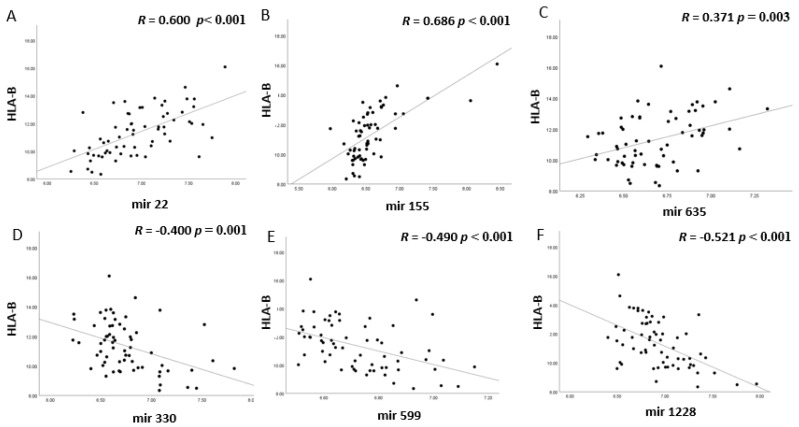
The expression levels of six different miRNAs (**A**–**F**) were compared to HLA-B mRNA levels in 64 Uveal Melanoma from Leiden. Positive and negative patterns are observed. *p* values were determined by Spearman correlation. *p* ≤ 0.05 was considered significant. (**A**) miR-22, (**B**) miR-155, (**C**) miR-635, (**D**) miR-330; (**E**) miR-599; (**F**) miR-1228 versus HLA-B expression.

**Figure 5:**
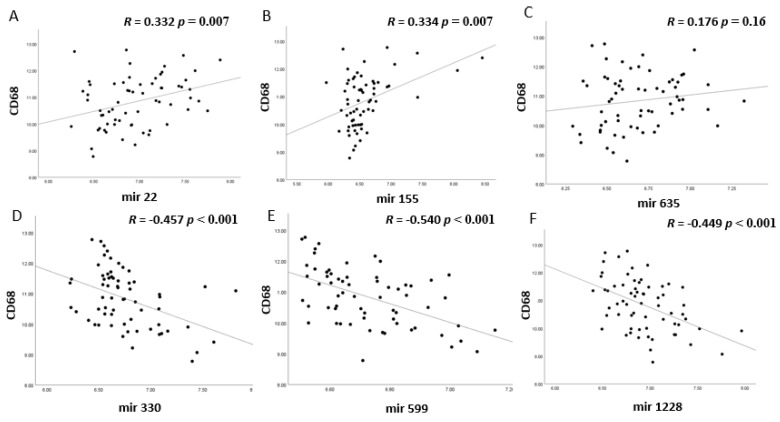
The expression levels of six different miRNAs (**A**–**F**) were compared to the mRNA levels of CD68, a macrophage marker, in 64 Uveal Melanoma from Leiden. *p* values were determined using Spearman correlation. *p* ≤ 0.05 was considered significant. (**A**) miR-22, (**B**) miR-155, (**C**) miR-635, (**D**) miR-330; (**E**) miR-599; (**F**) miR-1228 versus CD68 expression.

When looking at the correlation matrix, all four miRNAs (miR-22, miR-155, miR-635, miR-1276) that had shown a positive correlation with at least three of the four HLA probes, cluster together with the HLA probes, the T lymphocyte markers (CD3, CD4 or CD8) and the macrophage markers (CD68 and CD163), while this is not the case for the negatively-associated miRs; they cluster in an opposite pattern (Figure 3). The two opposing patterns are clearly visible in the correlation between the miRs with HLA-B (Figure 4), and macrophage marker CD68 (Figure 5): MiR-22, miR-155, and miR-635 show a positive correlation with HLA-B (*p* values ≤ 0.003) and MiR-22 and miR-155 with CD68 (both *p* values = 0.007), while miR-330, miR-599, and miR-1228 show negative correlations with HLA-B and CD68 (all *p* values ≤ 0.001).

As a control, we looked at the association between miRs of the Rotterdam cohort of 26 UM and the mRNA levels of infiltrating cells: in agreement with the prior findings, miR-155 was positively correlated with CD4 (*p* = 0.02), while miR-211 was negatively correlated with CD4 and CD8 and miR-125b2 with CD4 and CD163 (Table S4).

Of the four miRs in the Leiden cohort that had shown a positive correlation with HLA Class I and infiltrating leukocytes, three were present in the TCGA cohort: miR-155 was positively associated with CD4 (*R* = 0.336, *p* = 0.002), CD8 (*R* = 0.563, *p* < 0.001), CD68 (*R* = 0.277, *p* = 0.01) and CD163 (*R* = 0.365, *p* = 0.001); miR-22 was positively associated with CD8 (*R* = 0.296, *p* = 0.01) and CD163 (*R* = 0.254, *p* = 0.02) and not with CD4 (*R* = 0.142, *p* = 0.21) or CD68 (*R* = −0.105, *p* = 0.35), while miR-98 was only correlated with CD163 (*R* = 0.246, *p* = 0.03) and not with other markers: CD4 (*R* = 0.056, *p* = 0.62), CD8 (*R* = 0.162, *p* = 0.15), CD68 (*R* = −0.103, *p* = 0.36).

### 3.3. MiRNA Expression and HLA Class I Regulatory Factors and the Antigen-Loading Machinery

**Figure 6:**
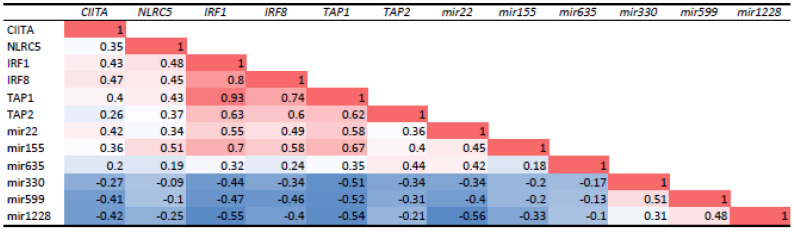
Correlation coefficients of the relation between several miRNAs and regulators of HLA expression and of members of the Antigen-Loading Machinery in the Leiden cohort. The expression of three miRNAs (miR-22, 155, 635) that are positively associated with HLA expression and of three (miR-330, 599, 1228) that are negatively associated with HLA expression were compared to Class I Regulatory Factors and members of the Antigen-Loading Machinery. The correlation coefficients based on Spearman correlations (*n* = 64) are shown. The intensity of colors indicates the strength of the correlation, with red indicating positive and blue negative correlations.

HLA expression is influenced by regulatory factors such as IRF1, IRF8, NLRC5, and CIITA, but also through the Antigen-Loading Machinery (ALM). We investigated whether there was an association between the miRNAs that were associated with HLA Class I and these regulatory factors (Figure 6). Indeed, miR-22 and miR-155 were positively associated to the ALM components TAP1 and TAP2, and the HLA regulatory factors CIITA, NLRC5, IRF1, and IRF8, while the opposite was found for miR330, miR-599, and miR-1228.

### 3.4. MiRNA Expression and Chromosome 3 Status

**Figure 7:**
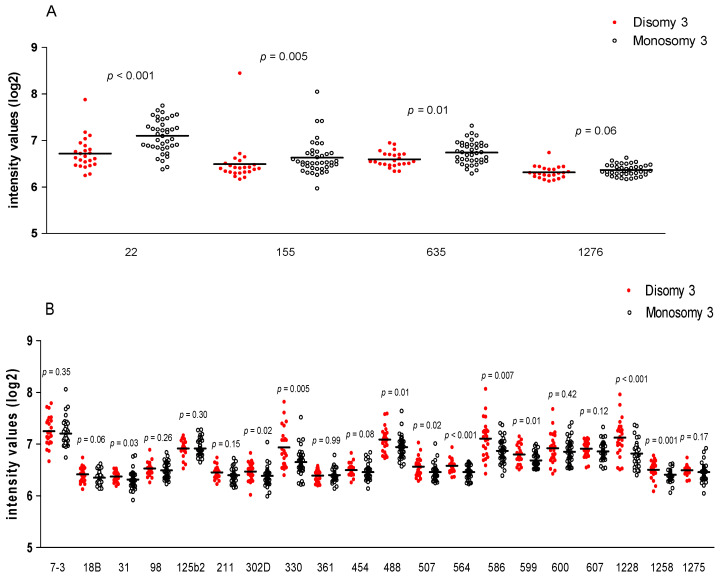
The expression of miRNAs in D3 (*n* = 24) and M3 (*n* = 40) tumours was compared. MiRNAs that are positively (**A**) or negatively (**B**) associated to HLA Class I are shown. Using a Mann-Whitney U test, *p* ≤ 0.05 was considered significant. MiRNA name is indicated as numbers. Horizontal bars indicate mean expression.

As we and others [10,11,12] have shown that an inflammatory infiltrate is associated with M3, we subsequently determined whether the differential expression of miRNAs was related to the tumour chromosome 3 status. Of the four miRNAs which were high in tumours with a high HLA expression and infiltrate, three (miR-22, miR-155, and miR-635) were significantly higher in M3 tumours than D3 tumours (Figure 7A). Of the 20 miRs that were negatively correlated to HLA Class I, ten showed a lower mean expression in M3 tumours than in D3 tumours (Figure 7B).

### 3.5. MiRNA Expression and BAP1 Expression

**Figure 8:**
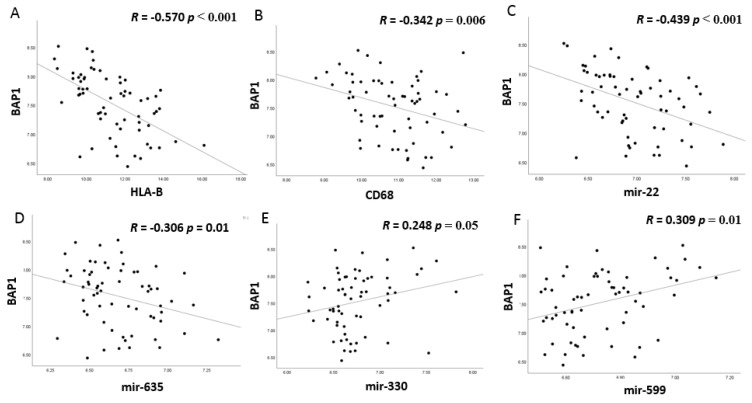
Correlation between BAP1 mRNA expression and HLA-B (**A**), CD68 (**B**), and examples of the two groups of miRNAs (**C**–**F**). *p* values were determined by Spearman correlation. *p* ≤ 0.05 was considered significant. (**A**) HLA-B, (**B**) CD68, (**C**) miR-22, (**D**) miR-635; (**E**) miR-330; (**F**) miR-599 versus BAP1 expression.

In addition to loss of chromosome 3, loss of expression of BAP1 is associated with increased inflammation in UM [13]. We compared mRNA expression levels of BAP1 with HLA expression and the presence of TAMs; BAP1 expression was negatively correlated with HLA-B (*p* < 0.001) and CD68 (*p* = 0.006) (see Figure 8). Moreover, BAP1 expression was negatively correlated to the pro-inflammatory miR-22, miR-155, and miR-635 (*p* < 0.001, *p* < 0.001, *p* = 0.01, respectively), and had a positive correlation with the anti-inflammatory miR-330, miR-599, and miR-1228 (*p* = 0.05, *p* = 0.01, and *p* = 0.001, respectively).

### 3.6. MiRNA Expression and Survival

**Figure 9:**
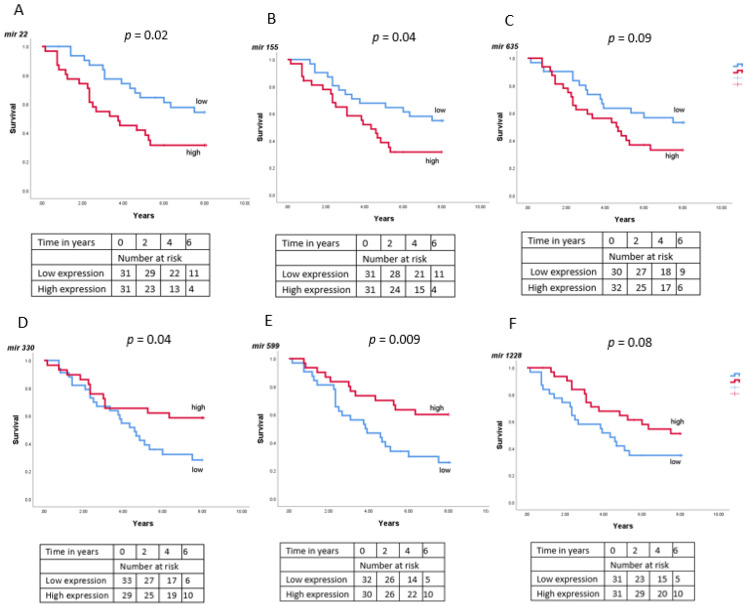
The relation between low and high miRNA expression and survival was determined in a group of 64 Uveal Melanoma patients from Leiden, using Kaplan-Meier survival curves (**A**–**F**). A log-rank test was used to determine significance. *p* ≤ 0.05 was considered significant. (**A**) miR-22; (**B**) miR-155; (**C**) miR-635; (**D**) miR-330; (**E**) miR-599; (**F**) miR-1228.

As we found associations between miRNA expression and loss of chromosome 3/BAP1 mRNA expression, we checked whether the levels of miRNAs were related to survival in UM patients (Figure 9).

According to Kaplan-Meier survival curves, high levels of miR-22 (*p* = 0.02) and miR-155 (*p* = 0.04) were related to decreased survival in UM patients while the opposite was found for miR-330 (*p* = 0.04) and miR-599 (*p* = 0.009). Differences with regard to miR-635 (*p* = 0.09) and miR-1228 (*p* = 0.08) did not reach statistical significance.

When we looked at the miRNA from the TCGA, we found that high levels of miR-22 (*p* = 0.003) and miR-155 (*p* = 0.004) were related to a decreased survival while this was not the case for miR-98 (*p* = 0.26).

## 4. Discussion

UM are defined by mutations which carry different risks of metastases. Most UM carry a mutation in the GNAQ or GNA11 gene, which do not contribute to prognostication [44,45,46]. EIF1AX and SF3B1 mutations occur especially in low-risk D3 tumours [47], while BAP1 mutations are observed in most high-risk M3 UM [13]. The tumours with M3/loss of BAP1 often show an inflammatory phenotype. However, despite all the different investigations regarding inflammation as a contributor towards metastasis in UM, it is still unclear how inflammation is regulated and how immune cells are recruited [39,48]. We therefore set out to explore which miRNAs are associated with inflammation in UM and whether their expression is related to chromosome 3/BAP1 loss. Robertson et al. previously identified different miRNA clusters in UM which were associated with chromosome 3 status and also suggested that expression of some miRNAs might be related to the immune environment [11]. Similar to previous studies [11,31,32,33,34,35], we observed a difference in miRs between low and high-risk UM.

One of the problems when looking at miRs is that different studies identify different miRs as being relevant: as stated in an extensive review by Aughton et al. [33], it should be noted that many studies looked at miRNAs in UM and hardly ever were the same miRNAs reported as significantly associated with an increased metastatic risk in UM. As they stated, differences may be due to differences in tissue sampling, classifications or miRNA detection systems.

As we were interested in relations between miRs and inflammation, we first tried to identify patterns. HLA Class I expression is a key component in the development of inflammation in the eye, an immune-privileged organ. In spite of this immune privilege, inflammation occurs in many intraocular UM. A study from our laboratory in 1996 already described positive correlations between HLA expression and the presence of infiltrating leukocytes such as CD3 and CD4 lymphocytes, monocytes/macrophages and NK cells in UM [49]. In addition, van Essen in 2016 found a positive association between HLA Class I, members of the Antigen Processing Machinery, such as TAP1, and the amount of lymphocyte and macrophage infiltration in UM [40]. He showed that mRNA expression correlated with immune-histochemical staining for HLA Class I, and we therefore used mRNA expression with four different HLA Class I probes to compare HLA expression with miRNA levels in the same tumours.

Out of the 125 miRNAs for which we had information from the Leiden cohort, we identified 24 that were associated with HLA Class I expression. Among these 24 miRNAs, we identified two clusters, one with a positive association with HLA Class I and infiltrating leukocytes, and the other cluster showing negative associations. Three of the four miRNAs (miR-22, 155, 635, and 1276) that were associated with a high HLA expression, were also related to high numbers of TILs and/or TAMs and were increased in tumours with M3. Most of the 20 miRNAs that showed an inverse correlation with HLA Class I expression also showed an inverse association with infiltrating leukocytes; ten of these miRNAs were decreased in tumours with M3. 

Several of the miRs have previously been associated with HLA expression or with inflammation. Aughton et al. [33] compared the information from several papers on miRNAs in uveal melanoma and only found a few that had been reported in multiple papers. They described that miRNA that were associated with an increased metastatic risk could either be upregulated or downregulated. Earlier, Worley et al. [31] had also shown that miRNA clustered into an upregulated and downregulated group, both related to prognosis. One of the upregulated miRs was miR-155 [33].

This miR is not only upregulated in UM, it is also increased in plasma of UM patients at the time of metastasis [50]. One possibility is that miR-155 functions as an immune stimulator: Mir-155 is known as an important regulator of the NFkB pathway in macrophages and thereby may modulate inflammatory responses [51]. We previously described that NFkB activity, one of the most important signaling pathways in inflammation, is increased in BAP1-negative UM [52]; here we show that miR-155 is elevated in M3 tumours which we know are mostly BAP1-negative. Further studies are needed in order to determine whether miR-155 plays a role in the regulation of NFkB in UM. Our data do not exclude the possibility that we are looking at the presence of miR-155 containing macrophages, as it is known that this miR is expressed in monocytes in peripheral blood and in CD68+ cells obtained from healthy brain tissue [53]. The association of miR-155 with TAM may therefore be due to its presence within these cells, thereby explaining its high expression in M3 UM, which are known to carry many macrophages [10]. The presence of infiltrating cells may influence the tumour cells’ HLA expression, as we previously described [40]. In a study involving epithelial ovarian cancer (another immune-privileged organ), miR-22 was similarly associated with the presence of an intra-tumoural immune infiltrate [54].

When we analysed which miRNAs were correlated with HLA expression, several strong positive correlations were observed, but, in addition, many other miRs showed a negative correlation. This group of miR also showed a negative correlation with the presence of TILs and TAMs, and with members of the Antigen Processing Machinery, such as TAP1. One of the downregulated miRNAs in high-risk M3 UM was miR-599. One study induced inflammation in endothelial cells by lipopolysaccharide (LPS) and found downregulation of mir-599 in endothelial cell injury, with upregulation of TNF, IL-6, ICAM-1, and VCAM-1 [55]. Overexpression of miR-599 led to the suppression of the inflammatory factors and downregulation of the JAK-STAT pathway known to be important in migration, proliferation, and inflammation by targeting ROCK1, a suppressor of inflammatory processes. Another miR with potential immunosuppressive capacity was miR-1228. In gastric cancer, miR-1228 was decreased in cell lines, and its induction led to a decrease of mesenchymal markers and invasiveness [56]. This decrease of miR-1228 was held responsible for NFkB upregulation.

That miR expression may play a role in HLA expression was demonstrated by the group of Seliger, which observed that in renal cell carcinoma, overexpression of specific miRNAs led to a reduction of HLA-G, enhancing NK-cell mediated cytotoxicity in vitro. Tumours expressing HLA-G had a significantly higher frequency of CD3+ and CD8+ T cells [57]. In cutaneous melanoma, TAP1 was the target of regulation by miRs. MiR-200a-5p was found to bind to the 3′untranslated region (UTR) of TAP1, and overexpression in a cell line was accompanied by a decrease in HLA-Class I surface expression, more so of HLA-B/C than HLA-A [58]. High levels of miR-200a-5p were associated with a shorter overall survival of the cutaneous melanoma patients. Mari et al. (2018) observed that in esophageal adenocarcinoma, miRs were able to regulate expression of TAP1 and HLA-Class I [59]. It looks as if a similar mechanism may play a role in a range of malignancies, including UM, but every malignancy may involve different miRs, and that some may be immune-suppressive, while others may have the opposite effect. We not only observed strong positive correlations between several miRNA and TAP1 (with miR-22, miR-155, and miR-635), but also identified many negative correlations, such as, e.g., with miR-330, miR-599, and miR-1228 (Table 1, Figure S1).

In UM, the group of three miRs were not only positively related to HLA Class I and TAP1 expression, but also with the HLA-transcription factors CIITA, NLRC5, IRF1, and IRF8. We suggest that a group of miRs suppress HLA expression and that during tumour progression (associated with addition of copies of chromosome 8q and loss of one chromosome 3 and loss of BAP1 expression) the HLA-suppressive miRNAs are lost and the stimulatory ones get expressed.

While affecting HLA expression, miRNAs may also function as oncogenes, affecting proliferation, and as regulators of inflammation, influencing the influx of inflammatory cells during tumour progression. Such a function has been attributed to e.g., miR-330, which has a tumour suppressive role in ovarian cancer, being able to downregulate MAPK signaling and ERK proteins [60], but also influencing inflammation: a forced expression of miR-330 led to the inhibition of oxidative stress and inflammation in macrophages [61].

Overall, we report two patterns for the expression of miRNA in UM, both related to the tumour chromosome/BAP1 expression status: one showing a relation between an increase in specific miRNAs and infiltrate, the other an opposite relationship. When looking at the miRNAs that are increased in UM that display inflammation, two options appear: as both miRNAs as well as leukocytes are present in a higher concentration in M3 tumours, it may be that we are looking at expression of miRNAs in infiltrating leukocytes. Another option is that loss of chromosome 3/BAP1 led to an upregulation of certain miRNAs in the UM cells themselves, which subsequently influenced the influx of inflammatory cells and led to upregulation of HLA antigens. It is clear that the genetic make-up of the tumour greatly influences miR expression and may thereby regulate HLA expression and inflammation.

Using the TCGA gene expression data, Sharma et al. [62] observed that a cluster of miRNAs was embedded in the 3′UTR region of the BAP1 gene. They analysed which miRNAs would best bind to BAP1 and its mutations. These BAP1-associated miRNAs affected at least 69 target genes. Among them were e.g., histone deacetylases (HDAC1, HDAC2, HDAC3 and HDAC4), and immune regulators such as TGFbeta1, TNF, and NFkB1, RELA and RELB. We have described associations between upregulation of the NFkB pathway and these molecules in UM with BAP1 loss [52]. Their miRNAs differed from the ones we identified.

The loss of one chromosome 3 has for a long time been associated with a worse prognosis in UM [1]. It has been shown that a mutation in BAP1 on chromosome 3 is similarly associated with a bad prognosis, leading to loss of BAP1 expression. We previously described an association between chromosome 3/BAP1 loss and inflammatory phenotype in UM [10,12,13], which has been confirmed by others [11,63,64]. We therefore propose that BAP1 loss may influence the presence of miRs, which may regulate inflammation. We propose a functional network of miRs, some of which have a pro-inflammatory role, as seen for miRs miR-22, miR-155, and miR-635, and some which have an anti-inflammatory function, or are just bystanders. However, both groups seem to be regulated by BAP1. It may be interesting to use the identified infiltrate-associated miRNAs to reduce local inflammation and decrease the level of cytokine production at the tumour site. Furthermore, it is important to look at other types of RNA: in addition to coding genes which may contribute to the development of UM, and miRNAs, non-coding genes that express non-translating RNAs such as lncRNA’s have also been shown to be involved in the pathogenesis of this disease. The long non-coding RNA *LINC00518,* controlled by MITF, has been shown to be involved in the progression of UM [65] while *RHPN1-AS1* was an inducer of migratory characteristics [66].

## 5. Conclusions

We have identified two patterns of miRNAs: a set of miRNAs are either related to an up- or to a downregulation of HLA Class I expression, TAP1, and an inflammatory infiltrate in UM, while both show a relation to chromosome 3 status and BAP1 expression. Specific miRNAs may therefore be regarded as immune-stimulatory miRNAs, while others act as immune-suppressants. The upregulated miRNAs may serve as therapeutic targets, while the downregulated miRs could be investigated for their potential to inhibit inflammation. Taken together, our study provides the basis to consider the miRNAs as important regulators of inflammation in UM, under the regulation of BAP1. 

## Figures and Tables

**Table 1:** Overview of correlations between different miRNAs and HLA-A, HLA-B and TAP1 in the Leiden cohort (*n* = 64), TCGA cohort (*n* = 80) and the Rotterdam cohort (*n* = 26). *r* = two-tailed Spearman correlation coefficient. *p* ≤ 0.05 considered significant. Significant correlations are indicated as bold. Negative associations are underlined.

miRNA	HLA-A			HLA-B			TAP1		
	Leiden (*n* = 64)	TCGA (*n* = 80)	Rotterdam (*n* = 26)	Leiden (*n* = 64)	TCGA (*n* = 80)	Rotterdam (*n* = 26)	Leiden (*n* = 64)	TCGA (*n* = 80)	Rotterdam (*n* = 26)
miRNA-22	**<0.001**	**0.003**	0.17	**<0.001**	**<0.001**	0.24	0.06	**0.002**	0.74
miRNA-155	**<0.001**	**<0.001**	0.08	**<0.001**	**<0.001**	0.07	**<0.001**	**<0.001**	**0.02**
miRNA-125-B2	**0.04**		**0.007**	**0.02**		**0.05**	0.15		**0.004**
miRNA-211	**0.004**		**0.05**	**0.04**		0.11	**0.002**		**0.005**

## Data Availability

Data from the Leiden cohort are accessible through GEO Series accession number GSE84976 (https://www.ncbi.nlm.nih.gov/geo/query/acc.cgi?acc=GSE84976). For the TCGA data see [11]. For the Rotterdam data see [35].

## Supplementary Materials

The following are available online at https://www.mdpi.com/article/10.3390/cancers13164020/s1, Figure S1: The expression levels of six different miRNAs were compared to the mRNA levels of TAP1 in 64 Uveal Mela-noma in the Leiden cohort, Table S1: Characteristics of patients and tumours in the Leiden cohort of 64 uveal melanoma used in the miRNA study. Table S2: Correlation between different miRNAs and HLA Class I in UM (n = 64). r = two-tailed Spearman correlation coefficient. *p* ≤ 0.05 considered significant. Shaded in blue: negative association; shaded in red: positive association. Only miRNAs were selected for further analysis that had significant associations with at least three of the four HLA probes. Table S3: Correlation between the miRNAs (which are not associated with HLA Class I) with TIL and TAM markers in UM (n = 64). r = two-tailed Spearman correlation coefficient. *p* ≤ 0.05 considered significant. Table S4: Correlation between miRNA gene expression and HLA Class I and infiltrate markers in a cohort of 26 UM tumours from Rotterdam; Spearman correlation, *p* ≤ 0.05 considered significant.
