# Supplementary material for: MiRNAs Correlate with HLA Expression in Uveal Melanoma: Both Up- and Downregulation Are Related to Monosomy 3
Source: Cancers (Basel). 2021 Aug 10;13(16):4020. doi: 10.3390/cancers13164020 (PMC8393554; doi:10.3390/cancers13164020)
Supplement: Supplementary file 1 [file cancers-13-04020-s001.zip › cancers-1324653-supplementary.pdf]

cancers

MDPI

Supplementary Materials: MiRNAs Correlate with HLA Expression in Uveal Melanoma: Both Up- and Downregulation Are related to Monosomy 3

Zahra Souri, Annemijn P.A. Wierenga, Emine Kiliç, Erwin Brosens, Stefan Böhringer, Wilma G.M. Kroes, Robert M. Verdijk, Pieter A. van der Velden, Gregorius P.M. Luyten and Martine J. Jager

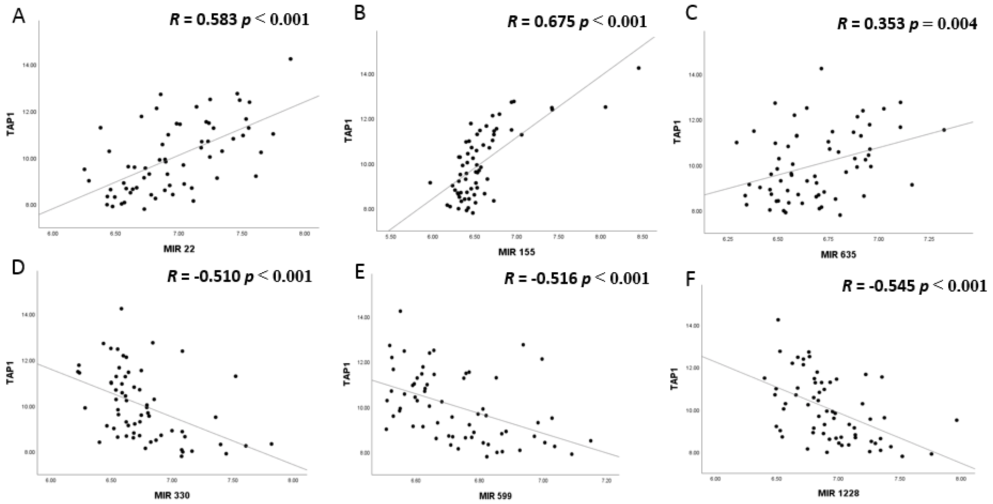

Figure S1. The expression levels of six different miRNAs (A-F) were compared to the mRNA levels of TAP1 in 64 Uveal Melanoma in the Leiden cohort. Three pro-inflammatory miRNAs (miR-22, 155 and 635) show positive associations while three anti-inflammatory miRNAs (miR-330, 599, and 1228) show negative associations. Spearman correlation. P≤0.05 was considered significant.

Table S1. Characteristics of patients and tumours in the Leiden cohort of 64 uveal melanoma used in the miRNA study.

Characteristics	Number of Cases	% of Cases
**Gender**		
Male	33	51%
Female	31	49%
**Age (Years) at Enucleation (SD)**		
≤60	30	47%
>60	34	53%
**Cell Type**		
Spindle	22	33%
Mixed/epithelioid	41	62%
**Largest Tumour Diameter (LBD) in mm**		
<13.0 (median)	27	42%
⩾13.0 (median)	37	58%
**Tumour Prominence in mm**		
<8.0 (median)	29	45%
⩾8.0 (median)	35	55%
**Ciliary Body Involvement**		
Not involved	40	62%
Involved	24	37%
**cTNM Stage**		
Stage I-IIB	37	58%
Stage IIIA-IIIC	25	39%
**Metastasis**		
No	26	41%
Yes	38	59%

Table S2. Correlation between different miRNAs and HLA Class I in UM (n = 64). r = two-tailed Spearman correlation coefficient. $p \leq 0.05$ considered significant and indicated as bold. Shaded in blue: negative association; shaded in red: positive association. Only miRNAs were selected for further analysis that had significant associations with at least three of the four HLA probes.

miRNA	HLA-A pr1		HLA-A pr2		HLA-A pr3		HLA-B	
	r	*p*	r	*p*	r	*p*	r	*p*
mir-let7-B-HG	-.038	.76	-.049	.70	-.102	.42	-.025	.84
mir-let7-G	-.091	.47	-.089	.48	-.090	.48	-.103	.42
mir-7-3-HG	-.302	**.01**	-.302	**.01**	-.275	**.03**	-.274	**.03**
mir-10-A	-.162	.20	-.063	.62	-.145	.25	-.176	.16
mir-17-HG	-.208	.10	-.095	.45	-.095	.46	-.070	.58
mir-18-B	-.235	.06	-.303	**.01**	-.255	**.04**	-.270	**.03**
mir-21	-.227	.07	-.194	.12	-.116	.36	-.122	.32
mir-22-HG	.569	**<.001**	.654	**<.001**	.565	**<.001**	.600	**<.001**
mir-25	-.191	.13	-.257	.04	-.199	.12	-.255	**.04**
mir-29-B1	-.213	.09	-.106	.41	-.181	.15	-.148	.24
mir-30-D	-.197	.12	-.153	.23	-.229	.07	-.159	.21
mir-31	-.324	**.009**	-.242	**.05**	-.367	**.003**	-.263	**.04**
mir-34-A	-.147	.24	-.095	.46	.011	.93	-.080	.53
mir-96	-.088	.49	.019	.88	-.061	.63	.028	.83
mir-98	-.339	**.006**	-.384	**.002**	-.263	**.04**	-.340	**.006**
mir-100-HG	-.296	.08	-.209	.10	-.180	.15	-.250	**.04**
mir-106-A	-.125	.32	-.104	.41	-.124	.33	-.107	.40
mir-125-B1	-.090	.48	-.042	.74	-.105	.41	-.108	.39
mir-125-B2	-.254	**.04**	-.300	**.02**	-.272	**.03**	-.288	**.02**
mir-129-2	.038	.76	-.052	.68	.007	.96	-.020	.87
mir-130-A	-.242	.05	-.201	.11	.007	.96	-.132	.30
mir-135-B	.000	1	-.008	.95	.010	.94	-.023	.86
mir-149	-.132	.30	-.073	.57	-.122	.34	-.109	.39
mir-155-HG	.536	**<.001**	.579	**<.001**	.619	**<.001**	.686	**<.001**
mir-181-C	-.142	.26	-.287	**.02**	-.171	.18	-.227	.07
mir-185	-.313	.01	-.217	.08	-.219	.08	-.282	**.02**
mir-194-1	-.154	.23	-.142	.26	-.159	.21	-.148	.24
mir-199-B	.025	.85	-.081	.52	-.024	.85	-.005	.97
mir-202	-.180	.15	-.128	.31	-.130	.31	-.164	.20
mir-203	.042	.74	-.079	.53	-.007	.96	-.007	.96
mir-211	-.351	**.004**	-.381	**.002**	-.354	**.004**	-.262	**.04**
mir-212	-.127	.32	-.142	.26	-.197	.12	-.133	.29
mir-215	-.085	.50	-.048	.70	-.033	.80	.048	.71
mir-218-2	.043	.74	.058	.65	.071	.58	.029	.82
mir-219-2	.363	**.003**	.229	.07	.133	.30	.250	**.05**
mir-221	-.067	.60	-.046	.72	-.089	.49	-.135	.29
mir-297	-.052	.68	-.048	.70	.017	.90	.020	.88
mir-300	-.096	.44	-.139	.27	-.293	**.02**	-.261	**.04**
mir-302-C	.157	.21	.029	.82	.100	.43	.094	.46
mir-302-D	-.322	**.01**	-.243	**.05**	-.269	**.03**	-.271	**.03**
mir-320-C1	.094	.46	.078	.54	-.003	.98	-.030	.81
mir-330	-.404	**.001**	-.541	**<.001**	-.415	**.001**	-.400	**.001**
mir-335	.011	.93	.067	.60	-.066	.60	-.081	.53
mir-342	-.012	.92	-.085	.51	-.093	.47	-.005	.97
mir-345	-.195	.12	-.198	.12	-.209	.10	-.190	.13
mir-361	-.229	.07	-.301	**.02**	-.285	**.02**	-.333	**.007**
mir-365-A	.129	.31	.065	.61	.153	.23	.037	.77
mir-369	-.094	.46	-.089	.48	-.062	.62	-.047	.71
mir-373	-.049	.70	-.149	.24	-.083	.52	-.106	.41
mir-382	.119	.35	.196	.12	.166	.19	.189	.13
mir-423	.153	.23	.192	.13	.094	.46	.172	.17
mir-429	.210	.10	.243	**.05**	.115	.36	.221	.08
mir-448	-.138	.28	-.052	.69	-.052	.69	-.058	.65
mir-450-A1	-.172	.17	-.160	.21	-.226	.07	-.209	.10
mir-454	-.357	**.004**	-.241	**.05**	-.237	.06	-.279	**.03**
mir-486	-.046	.72	-.108	.40	-.048	.70	-.161	.20
mir-488	-.343	**.005**	-.545	**<.001**	-.397	**.001**	-.377	**.002**
mir-489	.209	.10	.240	.06	.181	.15	.230	.07

**mir-496**	-.049	.70	-.076	.55	-.150	.24	-.121	.34
**mir-504**	-.084	.51	.036	.78	.022	.86	-.075	.56
**mir-505**	-.147	.25	-.156	.22	-.054	.67	-.049	.70
**mir-507**	-.415	**.001**	-.442	**<.001**	-.239	.06	-.333	**.007**
**mir-518-E**	-.015	.91	.101	.43	.146	.25	.121	.34
**mir-525**	-.098	.44	.048	.71	-.030	.81	-.028	.83
**mir-526-A2**	-.386	**.002**	-.214	.09	-.212	.09	-.244	**.05**
**mir-539**	-.272	**.03**	-.103	.42	-.229	.07	-.229	.07
**mir-541**	.136	.28	.161	.21	.102	.42	.154	.22
**mir-551-A**	-.053	.68	.001	.99	-.064	.62	-.096	.45
**mir-557**	-.043	.74	-.189	.13	-.135	.29	-.115	.37
**mir-558**	-.114	.37	-.084	.51	-.063	.62	-.065	.61
**mir-559**	.008	.95	.047	.71	-.063	.62	.072	.57
**mir-564**	-.306	**.01**	-.353	**.004**	-.308	**.01**	-.363	**.003**
**mir-568**	-.147	.24	-.195	.12	-.181	.15	-.133	.30
**mir-574**	.167	.18	.178	.16	.252	**.04**	.176	.16
**mir-576**	-.138	.28	-.039	.76	-.006	.96	-.006	.96
**mir-577**	.093	.47	.087	.49	.137	.28	.123	.33
**mir-586**	-.427	**<.001**	-.557	**<.001**	-.441	**<.001**	-.466	**<.001**
**mir-590**	-.129	.31	-.007	.96	-.070	.58	-.104	.41
**mir-593**	-.087	.50	-.166	.19	-.149	.24	-.204	.11
**mir-599**	-.386	**.002**	-.570	**<.001**	-.462	**<.001**	-.490	**<.001**
**mir-600-HG**	-.391	**.001**	-.310	**.01**	-.409	**.001**	-.372	**.002**
**mir-604**	-.139	.27	-.115	.36	-.117	.36	-.161	.20
**mir-607**	-.307	**.013**	-.409	**.001**	-.362	**.003**	-.352	**.004**
**mir-630**	.117	.36	.253	**.04**	.207	.10	.194	.12
**mir-631**	.030	.81	.084	.51	.056	.66	.036	.77
**mir-635**	.383	**.002**	.375	**.002**	.322	**.009**	.371	**.003**
**mir-637**	.080	.53	.170	.18	.075	.56	-.039	.76
**mir-638**	-.003	.98	.001	.10	.007	.96	.004	.97
**mir-639**	-.018	.89	-.026	.84	.008	.95	-.019	.88
**miR-640**	-.065	.61	-.144	.26	-.159	.21	-.105	.41
**mir-642-A**	-.141	.27	-.128	.31	-.210	.10	-.134	.29
**mir-645**	-.017	.89	.030	.81	.065	.61	-.070	.58
**mir-657**	-.074	.56	-.069	.59	-.184	.15	-.077	.54
**mir-708**	-.029	.82	.009	.94	-.031	.81	.016	.90
**mir-759**	.091	.47	.009	.94	.113	.37	.009	.94
**mir-760**	.155	.22	.159	.21	.145	.25	.167	.19
**mir-877**	-.005	.97	.110	.38	.102	.42	.092	.47
**mir-933**	-.158	.21	-.166	.19	-.209	.10	-.197	.12
**mir-939**	.140	.27	.085	.51	.172	.17	.162	.20
**mir-940**	.063	.62	-.017	.89	.036	.78	.037	.77
**mir-943**	-.086	.50	-.043	.74	-.059	.64	.031	.81
**mir-1208**	-.138	.28	-.183	.15	-.088	.49	-.145	.25
**mir-1224**	.065	.61	.198	.12	.220	.08	.138	.28
**mir-1228**	-.428	**<.001**	-.564	**<.001**	-.469	**<.001**	-.521	**<.001**
**mir-1237**	-.079	.53	.051	.69	.002	.99	.059	.64
**mir-1246**	-.005	.97	.077	.55	.073	.57	.024	.85
**mir-1247**	-.132	.30	-.109	.40	-.176	.16	-.120	.34
**mir-1253**	.025	.85	.111	.38	.094	.46	.109	.39
**mir-1258**	-.325	**.009**	-.420	**.001**	-.229	.07	-.332	**.007**
**mir-1267**	.041	.75	-.001	.10	-.001	.10	.023	.86
**mir-1269-A**	-.108	.40	.077	.54	.010	.94	-.078	.54
**mir-1271**	-.215	.09	-.151	.23	-.135	.29	-.180	.15
**mir-1275**	-.258	**.04**	-.197	.12	-.266	**.03**	-.249	**.05**
**mir-1276**	.129	.31	.242	**.05**	.299	**.02**	.249	**.05**
**mir-1277**	-.135	.29	-.076	.55	-.066	.60	-.108	.39
**mir-1281**	.081	.53	.117	.36	.140	.27	-.018	.88
**mir-1282**	-.078	.54	.064	.61	.009	.95	-.003	.98
**mir-1323**	-.059	.64	-.223	.08	-.163	.20	-.121	.34
**mir-1537**	-.124	.33	.073	.57	.025	.85	-.022	.86
**mir-1909**	.125	.32	.111	.38	.088	.49	.085	.50
**mir-1914**	-.123	.33	-.101	.43	-.071	.58	-.181	.15
**mir-1915**	-.121	.34	-.062	.63	-.019	.88	-.054	.67
**mir-2115**	.055	.67	-.034	.79	-.080	.53	-.061	.63

**mir-2116**	-.041	.75	.059	.64	-.170	.18	-.070	.58
**mir-2278**	-.022	.86	-.114	.37	-.075	.56	-.078	.59

Table S3. Correlation between the miRNAs (which are not associated with HLA Class I) with TIL and TAM markers in UM (n=64). r = two-tailed Spearman correlation coefficient. p ≤0.05 considered significant and indicated in bold.

miRNA	CD3E		CD4		CD8A		CD68		CD163	
	r	*p*	r	*p*	r	*p*	r	*p*	r	*p*
**miR-let7-B-HG**	.160	.20	-.067	.60	.068	.60	.188	.14	-.121	.34
**miR-let7-G**	-.092	.47	.061	.63	-.025	.85	.012	.93	.048	.71
**miR-10-A**	-.073	.56	-.033	.79	-.080	.53	-.179	.16	-.022	.86
**miR-17-HG**	-.010	.94	-.098	.44	.000	.10	.087	.49	-.095	.46
**miR-21**	-.132	.30	.057	.65	-.015	.91	-.096	.45	.042	.74
**mir-25**	-.183	.15	-.159	.21	-.106	.41	-.233	.06	-.197	.12
**mir-29-B1**	-.078	.54	-.140	.27	-.126	.32	.042	.74	-.028	.82
**mir-30-D**	-.151	.23	-.169	.18	-.215	.09	-.164	.20	-.195	.12
**mir-34-A**	-.199	.12	-.069	.59	-.026	.84	-.032	.80	-.041	.74
**mir-96**	-.040	.75	.057	.66	-.022	.86	.080	.53	-.023	.86
**mir-100-HG**	-.176	.16	-.276	**.03**	-.191	.13	-.191	.13	-.141	.27
**miR-106-A**	.012	.93	-.003	.98	.095	.45	-.036	.78	-.056	.66
**mir-125-B1**	-.073	.57	.080	.53	-.050	.70	-.116	.36	.045	.72
**miR-129-2**	-.103	.42	-.131	.31	-.186	.14	-.343	**.005**	-.031	.80
**mir-130-A**	-.011	.93	-.016	.90	-.022	.86	-.038	.76	.077	.54
**mir-135-B**	-.046	.72	.091	.47	.109	.39	.032	.80	.015	.90
**miR-149**	-.072	.57	-.097	.44	-.113	.37	.100	.43	-.035	.78
**mir-181-C**	-.064	.61	-.013	.92	-.208	.10	-.223	.08	.151	.23
**mir-185**	-.304	**.01**	-.265	**.03**	-.302	**.01**	-.298	**.02**	-.040	.76
**mir-194-1**	-.010	.94	-.193	.13	-.055	.67	.047	.71	-.250	**.05**
**mir-199-B**	.052	.68	-.249	**.05**	-.125	.32	-.179	.16	-.252	**.04**
**mir-202**	-.097	.44	-.010	.94	-.146	.25	-.221	.08	.084	.51
**mir-203**	.201	.11	.067	.60	.064	.61	-.026	.84	-.031	.81
**mir-212**	-.232	.06	-.125	.32	-.160	.21	-.220	.08	-.040	.75
**mir-215**	-.031	.81	-.269	**.03**	-.010	.93	-.059	.64	.022	.86
**miR-218-2**	.200	.11	.100	.43	.171	.18	.051	.69	.184	.14
**mir-219-2**	.254	**.04**	.344	**.005**	.113	.38	.229	.07	.230	.07
**mir-221**	-.210	.10	-.238	.06	-.200	.11	-.290	**.02**	-.067	.60
**mir-297**	-.006	.96	-.112	.38	.029	.82	-.036	.78	-.057	.65
**mir-300**	-.148	.24	-.029	.82	-.223	.08	-.141	.26	-.081	.53
**mir-302-C**	.236	.06	.057	.65	.239	.06	.139	.27	.093	.46
**mir-320-C1**	-.034	.79	.075	.56	-.026	.84	.064	.61	.101	.43
**mir-335**	-.024	.85	-.148	.24	-.042	.74	-.140	.27	-.154	.23
**mir-342**	.011	.93	-.154	.22	-.044	.73	-.330	**.01**	-.182	.15
**mir-345**	-.114	.37	-.146	.25	-.158	.21	-.112	.38	-.197	.12
**mir-365-A**	.079	.53	-.219	.08	-.006	.96	-.127	.32	-.094	.46
**miR-369**	-.299	**.02**	-.081	.52	-.100	.43	.020	.88	-.125	.32
**mir-373**	-.184	.14	-.153	.23	-.207	.10	-.282	**.02**	-.088	.49
**mir-382**	.401	**.001**	.157	.21	.326	**.01**	.270	**.03**	.138	.28
**mir-423**	.197	.12	.165	.19	.157	.22	.155	.22	-.149	.24
**mir-429**	.388	**.002**	-.047	.71	.171	.18	.272	**.03**	-.151	.23
**mir-448**	-.075	.56	-.234	.06	-.042	.74	-.036	.78	-.190	.13
**mir-450-A1**	-.119	.35	-.033	.80	-.122	.34	-.108	.40	-.222	.08
**mir-486**	-.314	**.01**	-.176	.16	-.259	.04	-.333	**.01**	-.001	.99
**mir-489**	.048	.70	-.029	.82	.104	.41	.093	.47	.096	.45
**mir-496**	-.041	.75	-.132	.30	-.215	.09	-.226	.07	-.189	.14
**mir-504**	.086	.50	-.023	.86	.030	.82	.103	.42	.091	.47
**mir-505**	-.029	.82	-.057	.65	-.044	.73	-.119	.35	-.052	.68
**mir-518-E**	.102	.42	.090	.48	.133	.29	.177	.16	.121	.34
**mir-525**	.011	.93	-.080	.53	.098	.44	.171	.18	.041	.75
**mir-526-A2**	-.219	.08	-.160	.21	-.011	.93	-.051	.69	-.142	.26
**mir-539**	-.207	.10	-.267	**.03**	-.128	.31	-.158	.21	-.190	.13
**mir-541**	.242	.05	.016	.90	.262	**.04**	-.004	.97	-.035	.78
**mir-551-A**	.001	.99	-.024	.85	-.112	.38	.022	.87	.069	.59
**mir-557**	-.189	.13	-.208	.10	-.143	.26	-.376	**.002**	-.191	.13
**mir-558**	-.103	.42	-.089	.49	-.054	.67	.043	.74	-.276	**.03**
**mir-559**	.200	.11	.038	.77	.091	.47	.215	.09	.052	.68

**mir-568**	.016	.89	-.165	.19	-.108	.39	-.212	.09	-.313	**.01**
**mir-574**	.074	.56	.158	.21	.063	.62	.067	.60	.180	.15
**mir-576**	-.183	.15	-.066	.60	.024	.85	-.263	**.04**	.082	.52
**mir-577**	.183	.15	-.059	.65	.084	.51	-.102	.42	.098	.44
**mir-590**	.044	.73	-.203	.11	.032	.80	.100	.43	.022	.86
**mir-593**	-.175	.17	-.143	.26	-.217	.08	-.157	.22	-.134	.29
**mir-604**	.199	.11	.017	.90	.155	.22	.018	.90	.283	**.02**
**mir-630**	.083	.51	.178	.16	.104	.41	.229	.07	.221	.08
**mir-631**	.229	.07	.035	.78	.008	.95	.060	.64	-.018	.89
**mir-637**	-.054	.67	-.253	**.04**	-.076	.55	-.069	.59	-.254	**.04**
**mir-638**	.032	.80	-.193	.13	-.032	.80	.088	.49	-.235	.06
**mir-639**	-.019	.88	.117	.36	.103	.42	.047	.71	-.018	.89
**miR-640**	-.009	.95	-.046	.72	.061	.63	-.156	.22	.029	.82
**mir-642-A**	.085	.50	.001	.99	.014	.91	.066	.60	.157	.22
**mir-645**	-.170	.18	.054	.67	-.012	.92	-.017	.89	-.035	.78
**mir-657**	.077	.54	-.066	.61	-.041	.75	.008	.95	-.144	.26
**mir-708**	-.060	.63	-.092	.47	.051	.69	-.116	.36	.011	.93
**mir-759**	-.066	.61	-.134	.29	.031	.81	-.042	.74	-.125	.32
**mir-760**	.141	.27	.290	**.02**	.125	.32	.169	.18	.359	**.004**
**miR-877**	-.025	.85	.043	.74	.153	.23	.259	**.04**	.093	.46
**mir-933**	.023	.85	-.106	.40	-.133	.30	-.071	.58	-.159	.21
**mir-939**	.205	.10	.106	.41	.223	.08	.104	.41	.109	.39
**mir-940**	.144	.25	.011	.93	.040	.75	.198	.12	.044	.73
**mir-943**	-.006	.96	-.053	.67	-.045	.72	-.069	.59	.074	.56
**miR-1208**	.052	.68	-.185	.14	.001	.99	-.336	**.01**	.052	.68
**miR-1224**	.157	.22	.161	.20	.262	**.04**	.085	.51	.227	.07
**miR-1237**	.145	.25	.138	.28	.156	.22	.245	**.05**	.242	**.05**
**miR-1246**	-.002	.99	-.135	.29	-.072	.57	-.008	.95	-.084	.51
**miR-1247**	-.149	.24	-.178	.16	-.105	.41	-.160	.21	-.230	.07
**miR-1253**	.059	.64	.140	.27	.032	.80	.227	.07	.144	.26
**mir-1267**	.107	.40	-.042	.74	.173	.17	-.013	.92	-.178	.16
**mir-1269-A**	-.011	.93	-.078	.54	.114	.37	.011	.93	.088	.49
**mir-1271**	-.204	.11	-.339	**.006**	-.217	.08	-.201	.11	-.169	.18
**mir-1277**	-.117	.36	.089	.48	.010	.94	-.088	.49	.201	.11
**mir-1281**	-.240	.06	-.117	.36	-.091	.47	-.277	**.03**	.159	.21
**mir-1282**	-.081	.52	.011	.93	.059	.64	.026	.84	-.034	.79
**mir-1323**	-.019	.88	-.104	.41	-.096	.45	-.003	.98	-.174	.17
**mir-1537**	.033	.79	.033	.79	.022	.86	.113	.37	.019	.88
**mir-1909**	.121	.34	.101	.43	.027	.83	.015	.91	.102	.42
**mir-1914**	-.065	.61	-.053	.68	-.044	.73	-.082	.52	-.066	.60
**mir-1915**	-.082	.52	-.116	.36	-.180	.15	.071	.58	-.132	.30
**mir-2115**	.043	.73	.053	.68	-.031	.81	-.110	.39	-.117	.36
**mir-2116**	.042	.74	.020	.88	-.024	.85	.240	.06	-.243	**.05**
**mir-2278**	.002	.99	-.127	.32	-.237	.06	-.175	.17	.020	.87

Table S4. Correlation between miRNA gene expression and HLA Class I and infiltrate markers in a cohort of 26 UM tumours from Rotterdam; Spearman correlation, $p \leq 0.05$ considered significant and indicated as bold.

miRNA	Descriptive		HLA-A		HLA-B		TAP1		CD4		CD8		CD163	
	**Mean**	**SD**	**R**	***p***	**R**	***p***	**R**	***p***	**R**	***p***	**R**	***p***	**R**	***p***
**mir-125B1_3p**	9	12	-.248	.29	-.116	.63	-.392	.09	-.247	.29	-.373	.10	-.420	.06
**mir-125b2_3p**	36	24	-.585	**.007**	-.456	**.05**	-.617	**.004**	-.473	**.03**	-.317	.17	-.441	**.05**
**mir-125b_5p**	1088	2358	-.525	**.02**	-.359	.12	-.620	**.004**	-.411	.072	-.360	.12	-.469	**.04**
**mir-155_5p**	94	100	.397	.08	.415	.07	.502	**.02**	.526	**.02**	.308	.19	.355	.12
**mir-18B_5p**	45	38	-.403	.08	-.156	.51	-.268	.25	-.144	.543	-.237	.31	-.271	.25
**mir-211_3p**	273	141	-.420	.06	-.316	.17	-.580	**.007**	-.524	**.02**	-.489	**.03**	-.397	.08
**mir-211_5p**	26040	14088	-.442	**.05**	-.364	.11	-.605	**.005**	-.594	**.006**	-.449	**.05**	-.403	.08
**mir-22_5p**	268	113	.317	.17	.275	.24	.080	.74	.066	.78	.105	.66	-.027	.91
**mir-31_3p**	9	12	-.180	.45	-.206	.38	-.080	.74	.160	.502	.159	.50	-.033	.89
**mir-31_5p**	75	76	-.077	.75	-.009	.97	.117	.62	.287	.22	.271	.25	-.036	.88
**mir-361_3p**	539	214	.395	.08	.389	.09	.499	**.02**	.187	.43	.513	**.02**	.149	.53
**mir-361_5p**	1351	515	-.314	.17	-.346	.13	-.392	.09	-.548	**.01**	-.283	.23	-.302	.19
**mir-454_3p**	202	43	.006	.98	.042	.86	.111	.64	-.157	.51	-.002	.99	-.152	.52
**mir-507**	65	117	-.200	.40	-.111	.64	-.210	.37	-.136	.57	-.012	.96	-.267	.25
**mir-7_1_3p**	55	27	-.414	.07	-.227	.34	-.411	.07	-.276	.24	-.191	.42	-.205	.39
**mir-98_3p**	23	7	-.239	.31	-.223	.35	-.432	.06	-.351	.13	-.136	.57	-.319	.17
**mir-98_5p**	270	64	.388	.09	.389	.090	.120	.61	.011	.96	.196	.41	-.023	.92
